# Long-Term Denervated Rat Schwann Cells Retain Their Capacity to Proliferate and to Myelinate Axons *in vitro*

**DOI:** 10.3389/fncel.2018.00511

**Published:** 2019-01-07

**Authors:** Tessa Gordon, Patrick Wood, Olawale A. R. Sulaiman

**Affiliations:** ^1^Division of Neuroscience, Faculty of Medicine, University of Alberta, Edmonton, AB, Canada; ^2^The Miami Project to Cure Paralysis/Department of Neurological Surgery, University of Miami School of Medicine, Miami, FL, United States; ^3^Department of Neurosurgery, Ochsner Medical Center, New Orleans, LA, United States

**Keywords:** Schwann cells, nerve transection, myelination, SC proliferation, neurites, nerve injury

## Abstract

Functional recovery is poor after peripheral nerve injury and delayed surgical repair or when nerves must regenerate over long distances to reinnervate distant targets. A reduced capacity of Schwann cells (SCs) in chronically denervated distal nerve stumps to support and interact with regenerating axons may account for the poor outcome. In an *in vitro* system, we examined the capacity of adult, long-term denervated rat SCs to proliferate and to myelinate neurites in co-cultures with fetal dorsal root ganglion (DRG) neurons. Non-neuronal cells were counted immediately after their isolation from the distal sciatic nerve stumps that were subjected to acute denervation of 7 days or chronic denervation of either 7 weeks or 17 months. Thereafter, equal numbers of the non-neural cells were co-cultured with purified dissociated DRG neurons for 5 days. The co-cultures were then treated with ^3^H-Thymidine for 24 h to quantitate SC proliferation with S100 immunostaining and autoradiography. After a 24-day period of co-culture, Sudan Black staining was used to visualize and count myelin segments that were elaborated around DRG neurites by the SCs. Isolated non-neural cells from 7-week chronically denervated nerve stumps increased 2.5-fold in number compared to ~2 million in 7 day acutely denervated stumps. There were only <0.2 million cells in the 17-week chronically denervated stumps. Nonetheless, these chronically denervated SCs maintained their proliferative capacity although the capacity was reduced to 30% in the 17-month chronically denervated distal nerve stumps. Moreover, the chronically denervated SCs retained their capacity to myelinate DRG neurites: there was extensive myelination of the neurites by the acutely and chronically denervated SCs after 24 days co-culture. There were no significant differences in the extent of myelination. We conclude that the low numbers of surviving SCs in chronically denervated distal nerve stumps retain their ability to respond to axonal signals to divide and to elaborate myelin. However, their low numbers consequent to their poor survival and their reduced capacity to proliferate account, at least in part, for the poor functional recovery after delayed surgical repair of injured nerve and/or the repair of injured nerves far from their target organs.

## Introduction

The Schwann cells (SCs) in the peripheral nervous system support nerve regeneration after nerve injury in contrast to the oligodendrocytes in the central nervous system that do not (Cajal, 1928; Bunge, 1994; Fenrich and Gordon, 2004; Toy and Namgung, 2013). Yet, functional recovery is suboptimal after peripheral nerve injuries in patients who have sustained injury to large nerve trunks such as the brachial plexus (Sunderland, 1978; Kline and Hudson, 1995). The prevailing view is that this failure in recovery is due to the irreversible degeneration of chronically denervated muscles and their replacement with fat during nerve regeneration (Sunderland, 1978; Kline and Hudson, 1995). This is despite the early conclusions by Holmes and Young (1942) that there are “various factors, in addition to atrophy of the end-organs, which are likely to reduce the effectiveness of recovery when suture is made after a long delay” and the later conclusion that “Most likely, multiple mechanisms contribute to this phenomenon” (of diminished recovery of muscle mass and integrated motor function), “which is sometimes referred to as ‘irreversible denervation atrophy”’ (Kobayashi et al., 1997). Peripheral nerves regenerate at 1–3 mm/day, resulting in delays of months and, in humans, even years before regenerating nerves reach their denervated targets (Fu and Gordon, 1997). Gutmann and Young (1944) reported that denervated rat neuromuscular junctions progressively deteriorate after delayed nerve surgery. The authors attributed poor functional recovery after such a delay to an irreversible inability of regenerating nerves to reinnervate chronically denervated neuromuscular junctions. However, more recent studies have demonstrated that the progressive failure of even freshly injured (axotomized) neurons to successfully regenerate their axons through chronically denervated distal nerve stumps is the major factor accounting for progressive decline in functional recovery (Fu and Gordon, 1995a; Vuorinen et al., 1995; Sulaiman and Gordon, 2000; Gordon et al., 2003, 2011) and the recovery of denervated muscle mass (Kobayashi et al., 1997). Chronically denervated muscles do accept reinnervation despite their declining satellite population and the consequent incomplete recovery of reinnervated muscle fiber size and muscle wet weight (Fu and Gordon, 1995a; Kobayashi et al., 1997; Sulaiman and Gordon, 2000; Jejurikar et al., 2002; Gordon et al., 2011). Hence, changes in the microenvironment of the chronically denervated nerve stumps provide, at least in part, the explanation for the progressive failure of axonal regeneration through the nerve stumps and to the denervated muscles.

The specific changes in the microenvironment of the denervated distal nerve stump accounting for poor axonal regeneration, are not understood. Regeneration is fostered in acutely injured peripheral nerves by the functional, structural, and molecular changes occurring in the denervated SCs in the distal stumps. Denervated SCs take an active role in the phagocytosis of myelin, a role that is taken over by macrophages that infiltrate the denervated nerve stump after 3 days (Gibson, 1979; Avellino et al., 1995; reviewed by Gordon, 2015). The SCs proliferate and extend long processes across the injury site within the first 10 days after injury (Salonen et al., 1988; Son and Thompson, 1995; Witzel et al., 2005). They also participate in the development of reversible and organized endoneurial structures that include the synthesis and positioning of collagen fibrils close to the laminin-containing basal lamina of the SCs and the formation of fascicle-like structures by the endoneurial cells (Salonen et al., 1987a,b, 1988).

The early changes in the denervated SCs include the switch in their phenotype from myelinating to a growth supportive one, the SCs downregulating genes that transcribe myelin associated proteins and glycoproteins and upregulating hundreds of growth-supportive genes reciprocally (De Leon et al., 1991; El Soury et al., 2018). The latter include genes that are responsible for ribonuclear RNA metabolic processes which contribute to the synthesis and production of new proteins and molecules required for Wallerian degeneration and nerve regeneration (El Soury et al., 2018). The many growth associated genes include those that transcribe transcription factors, c-jun and Notch, neurotrophic factors (reviewed by Jessen and Mirsky, 2016; Gordon and Borschel, 2017), soluble neuregulin 1 (NRG1) type I and type II isoforms, the NRG1 receptor ErbB2/3 (Carroll et al., 1997; Audisio et al., 2008; Gambarotta et al., 2013; Ronchi et al., 2016), and Erbin, an ErB2 interacting protein required for remyelination (Liang et al., 2012). The neurotrophic factors include nerve growth factor (NGF), brain- and glial-derived neurotrophic factors, and pleiotrophin and the receptors include p75 and truncated trk receptors for the neurotrophic factors (Meyer et al., 1992; Funakoshi et al., 1993; Naveilhan et al., 1997; Hoke et al., 2002, 2006; Mi et al., 2007; Brushart et al., 2013). These early changes in the denervated SC phenotype are important in promoting axonal regeneration (Fu and Gordon, 1997; Boyd and Gordon, 2003; El Soury et al., 2018).

The decline in the capacity of chronically denervated SCs to support nerve regeneration is, at least in part, explained by the transient nature of neurotrophic factor and receptor upregulation (Boyd and Gordon, 2003; Gordon, 2015). Their upregulated gene expression declines within a month of denervation (Fu and Gordon, 1997; You et al., 1997; Hoke et al., 2002, 2006; Brushart et al., 2013), as does the upregulation of the erbB2/3 receptors (Li et al., 1997; Hall, 1999) required for the proliferation of denervated SCs (Carroll et al., 1997; Gambarotta et al., 2013). Importantly, the chronically denervated SCs atrophy and decline in numbers: progressive SC atrophy and loss with disruption of their basement membranes and shrinkage of their columns, have been reported in rat and rabbit hindlimb nerves over periods of 1 to 26 months (Weinberg and Spencer, 1978; Salonen et al., 1987a,b; Röyttä and Salonen, 1988; You et al., 1997; Bradley et al., 1998). SC numbers identified with S-100 immunoreactivity, declined from a peak of ~3.5 times that of normal nerves at 4 weeks to the relatively low levels seen in intact nerves by 30 and 50 weeks after chronic sciatic nerve stump denervation in adult Wistar rats (Salonen et al., 1988).

In the present study, we evaluated the long-term survival of chronically denervated SCs and their capacities for proliferation and for myelination in response to contact with neurites. We provide the first quantitative evaluation of survival of very long-term (17 months) survival of chronically denervated SCs and demonstrate their sustained capacity to proliferate and to myelinate axons.

## Materials and Methods

### Ethical Approval

All surgical procedures and perioperative care measures were performed with strict accordance with the National Institutes of Health guidelines. All the procedures were reviewed and approved by the University of Alberta animal care committee following the Canadian Council of Animal Care (CCAC) guidelines. Rat embryos from pregnant females were removed on the 15th day of gestation for dissection of dorsal root ganglia (DRGs) for isolation of the DRG sensory neurons. All the procedures of removal and isolation of DRG neurons were carried out in Miami and were reviewed and approved by the Miami Animal Care and Use Committee.

### Animals

Adult female Sprague-Dawley rats (180–200 g body weight, *n* = 20) were used in the experiments in which hindlimb nerves were transected and ligated *in vivo* prior to removal of the denervated distal nerve stumps. Pregnant rats (*n* = 2) were used to remove 8–14 embryos each on the 15th day of gestation.

### Materials

Cell culture reagents were purchased: Dulbecco’s Modified Eagle Medium (DMEM) and Neurobasal medium and B27 supplement from Gibco, 0.05% collagenase from Worthington, 0.25% dispase from Boehringer-Mannheim, fetal bovine serum (FBS) from Hyclone, partially purified NGF from ProspectBio, trypsin from Worthington, TRL3, NTB2 emulsion from Kodak, mouse laminin from Collaborative Research, Inc., and rabbit anti-S100 antibody from Dako Corporation.

### Anesthetic Protocols, Nerve Surgeries and Monitoring

All surgical procedures of sciatic nerve transection and ligation and the dissection of DRGs were made under anesthesia and using sterile surgical technique. Surgical anesthesia of the rats was induced with an intraperoneal injection of sodium pentobarbital (30 mg/kg).

#### *In vivo* Sciatic Nerve Transection of the Sciatic Nerves and Ligation of the Nerve Stumps

**Figure 1:**
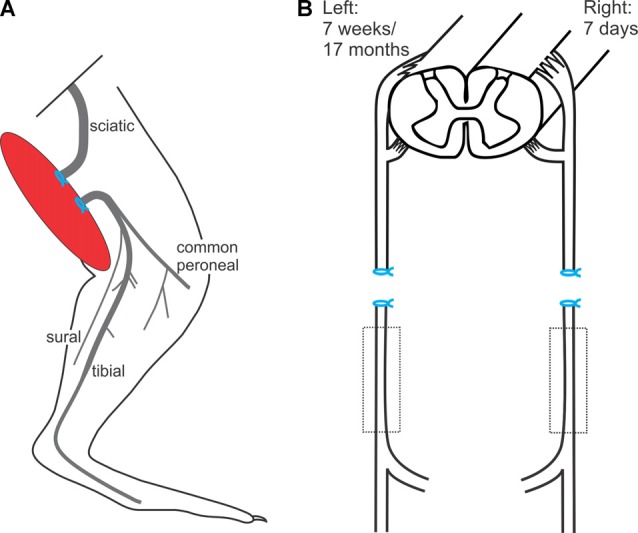
*In vivo* sciatic nerve transection and ligation to denervate Schwann cells (SCs) in the distal nerve stump. **(A)** Under surgical anesthesia and using aseptic precautions, the left sciatic nerve was exposed, cut, and the proximal and distal nerve stumps ligated and sutured to nearby innervated muscles to prevent axonal regeneration and reconnection for 7 weeks or for 17 months, the experimental groups of chronic distal nerve denervation in the left hindlimb. **(B)** In a second aseptic surgery, 7 weeks or 17 months later, the rats were again anesthetized and the right sciatic nerve was cut and ligated and again, the nerve stumps were sutured to innervated muscle in order to prevent regeneration for 7 days.

The left hindlimb was shaved and a lateral skin incision was made. The intermuscular septum between the biceps femoris and the vastus lateralis muscles was divided and the sciatic nerve identified. The sciatic nerve was freed using blunt dissection in order to cut the nerve sharply with scissors. The proximal and distal nerve stumps were ligated with 4.0 silk and sutured to nearby innervated muscles in order to prevent reconnection and subsequent axonal regeneration (Figure 1), as previously described (Fu and Gordon, 1995a,b; Sulaiman and Gordon, 2000; Gordon et al., 2011). The overlying muscle was closed and the skin incision on the thigh was closed in layers. The rats were placed under heat lamps and their recovery monitored.

In a second aseptic surgery, 7 weeks or 17 months later, the rats were again anesthetized, and the contralateral right sciatic nerve was cut and ligated. Again, the nerve stumps were sutured to innervated muscle in order to prevent regeneration for 7 days (Figure 1B).

#### Monitoring for Evidence of Nerve Regeneration

In a third aseptic surgical procedure, the skin was shaved on both hindlimbs and then opened to expose, bilaterally, the sciatic nerve and it’s tibial (TIB), common peroneal (CP) and sural nerve branches. An aseptic bipolar electrode attached to a Grass stimulator was used to deliver supramaximal stimuli at 10 Hz to the proximal sciatic nerve stump, followed by the distal stumps of the sciatic nerve and its branches to ensure that nerve regeneration had not occurred. No muscle contractions were observed in any of the rats confirming that the distal nerve stumps of the sciatic nerves were acutely denervated for 7 days and chronically denervated for 7 weeks and 17 months.

#### Surgical Removal of Sciatic, TIB, CP, and Sural Distal Nerve Stumps

In the same third surgical procedure, the chronically denervated (experimental) and the contralateral acutely denervated (control) distal stumps of the sciatic nerve and its branches in the left and right hindlimbs, respectively, were removed and placed in Belzer’s solution at 4°C for shipment to Miami for nerve dissociation, isolation of SCs, and preparation of co-cultures with DRG neurons.

#### Dissection of Dorsal Root Ganglia (DRGs) From Rat Embryos

Pregnant rats were deeply anesthetized with ether to allow surgery to remove embryos on the 15th day of gestation as previously described in detail (Kleitman et al., 1988). The DRGs from each of the 8–14 embryos were carefully dissected free and dissociated to establish DRG sensory neuron cultures (see below). The mothers were euthanized by exsanguination.

### *In vitro* Cellular Dissociations and Co-culture

#### Non-neuronal Cells From Denervated Nerve Stumps

Using sterile procedure, the epineurium was removed from the dissected distal nerve stumps of the sciatic nerve and the CP, TIB and sural nerve branches which had been chronically denervated for 7 weeks and 17 months (in the left experimental hindlimb or acutely denervated in the right control hindlimb for 7 days. Because the chronically denervated nerves were shrunken, especially those after the lengthy 17-month period of chronic denervation, the nerves for each of the three periods of nerve stump denervation were pooled for dissociation of their denervated non-neuronal cells, i.e., 10 nerves each were pooled after 7 weeks and 17 months of chronic distal nerve stump denervation and 20 nerves were pooled after 7 days of acute distal nerve stump denervation.

The nerves were incubated in culture medium containing 0.05% collagenase, 0.25% dispase solution in DMEM, and 10% FBS (Medium 1), for 18 h at 35–37°C. The fascicles were rinsed free of collagenase/dispase with DMEM + 10% FBS. They were then dissociated into a single cell suspension by gentle trituration, using a glass micropipette with a tip diameter of 0.3–0.5 mm. The non-neuronal cells were harvested and re-suspended in Neurobasal medium with 2% B27 supplement and partially purified NGF at 50 units/ml (hereafter referred to as Medium 2). The cells were then counted and thereafter, their capacities to proliferate and to myelinate isolated DRG neurons were determined.

#### DRG Neurons From Rat Embryos

DRGs dissected from rat embryos on the 15th day of gestation were sequentially: (1) incubated with 0.25% trypsin in calcium and magnesium—free Hank’s Balanced Salt Solution (Medium 3) for 1 h at 35–37°C on a slowly rotating shaker; (2) rinsed free of trypsin with L-15 medium containing 10% heat-inactivated FBS; and (3) dissociated by gentle trituration using a glass pipette with a tip diameter of 0.3–0.5 mm. The dissociated DRG cells were then harvested and re-suspended in Medium 2. The DRG cell suspension was plated at a density of 20,000 cells in a volume of two drops onto collagen-coated clear coverslips, 22 mm in diameter (Kleitman et al., 1988) and placed into 25 mm clear mini-dishes that were incubated in a 5% CO_2_ atmosphere at 37°C.

A day after plating, the DRG neuron cultures were flooded with 10 drops of the Medium 2 that was supplemented with fluorodeoxyuridine (FdU) and uridine, both at a concentration of 10 μM (antimitotic Medium 2). This antimitotic treatment was repeated on days 8 and 15 of culture. Thereafter, the antimitotic agents were excluded from the culture medium (Medium 2), the Medium 2 (without FdU and uridine) being used to wash out the antimitotic agents (on days 19, 21, 26, 28, and 33, and 42). This washing ensured the depletion of antimitotic agents from the culture environment of the DRG neurons prior to their co-culture with non-neural cells from the acutely and chronically denervated distal nerve stumps.

#### Co-culture of DRG Neurons and Non-neuronal Cells From Denervated Nerve Stumps

The DRG neurons in each mini-dish were plated with 100,000 non-neuronal cells on the same day as the dissociation of the non-neuronal cells from the chronically (7 weeks and 17 months) and acutely (7 days) denervated distal nerve stumps.

### Experiments

#### Cellular Counts

Small aliquots of non-neuronal cell suspensions, prepared in Medium 2 of each of the 7-week and 17-month chronically denervated nerve stumps and from the 7-day acutely denervated distal nerve stumps, were fixed in 4% paraformaldehyde in 0.05 M sodium phosphate buffer, pH 7.4 for 15 min at room temperature and stained with Hoechst 33342 dye (10 μM). The cells were visualized by fluorescence microscopy, counted in a hemocytometer, and the total cell numbers calculated. The resulting bright staining of cell nuclei allowed the unambiguous recognition of the cells even when the suspensions were heavily laden with myelin debris.

#### Cellular Proliferation Assay

The non-neuronal cell suspensions were diluted with Medium 2 to 100,000 cells/ml and plated onto cultures of isolated DRG neurons at 1.0 ml per culture. Control neuronal cultures received 1.0 ml of Medium 2 without suspended cells. For maintenance, the co-cultures were re-fed three times each week with Medium 2.

After 5 days of co-culture, the non-neuronal cells in each of four cultures from the 7-day acutely denervated control group and the 7-week and 17-month chronic denervation experimental groups were labeled for 24 h with ^3^H-thymidine (0.5 μCi/ml) in Medium 2. The cultures were then rinsed and fixed with 4% paraformaldehyde in 0.1 M sodium phosphate buffer, pH 7.4 for 10 min at room temperature, permeabilized with 0.2% Triton X-100 in the fixative for another 10 min at room temperature, and prepared for immunostaining by blocking (30 s at room temperature) in a solution of 50% heat-inactivated goat serum in L-15 medium. The co-cultures were treated with rabbit anti-S100 antibody at 1:100 dilution in L-15/10% heat-inactivated goat serum for 30 min at room temperature, rinsed, and treated with fluorescein-conjugated goat anti-rabbit secondary antibody at 1:100 dilution in L-15/10% heat-inactivated goat serum for an additional 30 min at room temperature. The cultures were rinsed with buffer, distilled water and ethanol, dried and mounted on glass slides, cell side up, with DPX mountant. The slides were dipped in NTB2 emulsion and processed for autoradiography (Wood and Bunge, 1975). The counts of the S-100 positive cells were made directly under microscopic view and not made on photographic images with their possible distortions. A proliferation index was defined as the number of the S-100 positive SCs that were labeled with ^3^H-thymidine expressed as a percentage of the total number of S-100 positive SCs.

#### Myelination Assay

Six co-cultures per group of DRG neurons and non-neuronal cells isolated from the chronically denervated sciatic, TIB, CP, and sural nerve stumps, were maintained for 24 days in Medium 2 that was supplemented with 10 ng/ml of mouse laminin. Control cultures of DRG neurons without added non-neuronal cells were kept in parallel. All cultures were fixed overnight in FIX at 4°C, osmicated for 1 h in 0.1% osmium tetroxide in 0.1 M sodium phosphate buffer, pH 7.4, dehydrated to 70% ethanol in water and stained for 1 h in 0.7% Sudan Black in 70% ethanol in water (Kleitman et al., 1988). The cultures were rehydrated and mounted in glycerin jelly on glass slides. A quantitative measurement of myelination in each culture was made by counting the number of myelin segments crossing the equator of the cultures, using an eyepiece containing a single line horizontally bisecting the field of view at 200× magnification.

### Statistics

Statistical analysis was conducted using an ANOVA with the Tukey’s *post hoc* test (confidence interval = 95%). Comparisons were made between the experimental chronically denervated (7 weeks and 17 months) distal nerve stump of the cut and ligated left sciatic nerve and the control acutely denervated (7 day) distal nerve stump of the cut and ligated right sciatic nerve (Figure 1).

## Results

### The Number of Non-neuronal Cells in Peripheral Nerve Stumps Decline With Time After Chronic Denervation

The nuclei of the non-neuronal cells in suspension were unambiguously identified with Hoechst stain and their numbers counted in a hemocytometer to calculate their total cell numbers. The bright staining of cell nuclei allowed the unambiguous recognition of cells even when the suspensions were heavily laden with myelin debris.

**Figure 2:**
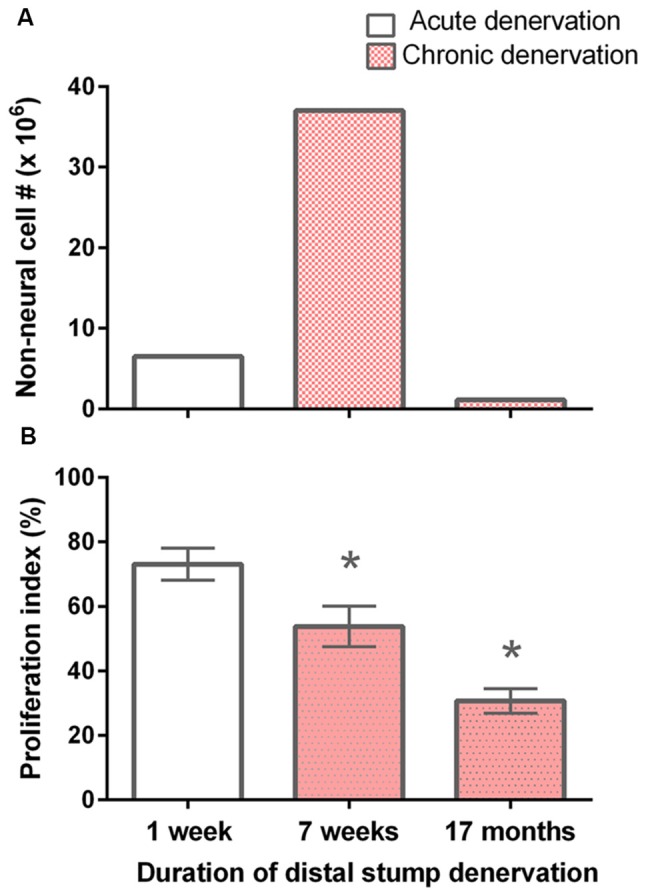
Decline in the number of non-neuronal cells and the proliferative capacity of S-100 positive SCs with chronic denervation. **(A)** Non-neuronal cells isolated from acutely (7 days) and chronically (7 weeks and 17 months) denervated distal nerve stumps of the sciatic nerve and its tibial (TIB), common peroneal (CP), and sural nerve branches, namely control acute and experimental chronically denervated distal nerve stump form the right and left hindlimbs, respectively. The cells were counted in small aliquots of single cell suspensions. **(B)** The mean (± SE) of the proliferation index. The index is the number of S-100 positive SCs that, in co-cultures with isolated dorsal root ganglion (DRG) neurons for 5 days, were labeled with ^3^H-thymidine and hence were dividing, expressed as a percentage of the total number of S-100 positive SCs. The proliferation indices at 7 days, 7 weeks and 17 months were significantly different from one another as indicated by *’s (*p* < 0.025).

The total numbers of non-neuronal cells from the experimental 7-week denervated distal nerve stump from the left hindlimb were >4 × 10^6^ cells as compared to <2 × 10^6^ cells in the acutely 7 day denervated distal stump from the right hindlimb in each rat (Figure 2). This increase was not sustained, the numbers after 17 months chronic nerve stump denervation falling to ~10% of those numbers enumerated in the control 7-day acutely denervated distal nerve stumps. The increase of non-neuronal cells after chronic denervation at 7 weeks as compared to the numbers after acute denervation at 7 days, likely include fibroblast-like and mast cells that also accumulate in the denervated nerve stump (Salonen et al., 1988; Ronchi et al., 2017) in addition to proliferating SCs in the nerve stumps during the Wallerian degeneration period of ~4 weeks (Salonen et al., 1988; You et al., 1997). The chronic denervation of the distal nerve stumps was controlled for the age and weight of the rats because the acutely and chronically denervated non-neuronal cells were dissociated from left and right hindlimbs, respectively, from the same rats.

### Chronically Denervated Schwann Cells Retain Their Capacity to Proliferate, Albeit at a Reduced Rate

**Figure 3:**
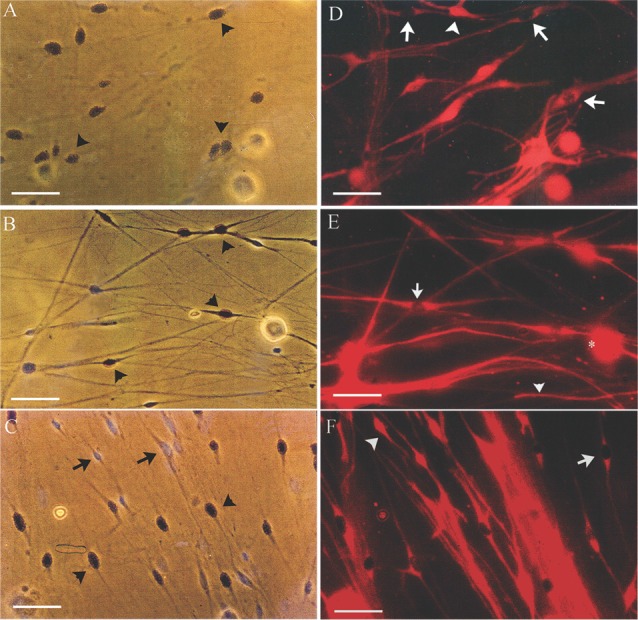
Many SCs in the nerve stump distal to the site of sciatic nerve transection and ligation are in an active proliferative state after acute as well as after chronic denervation, at least for up to 7 weeks. Phase-contrast autoradiographs of isolated acutely (**A,D**: 7 days) and chronically (**B,E**: 7 weeks and **C,F**: 17 months) denervated SCs from right and left hindlimbs, respectively. **(A–C)** Phase-contrast of the SCs that were exposed to ^3^H-thymidine for 24 h to identify dividing cells, 5 days after co-culture with DRG neurons. Many of the SCs incorporated ^3^H-thymidine (arrowhead) demonstrating SCs in an active proliferative state in the acute phase of denervation **(A)** and up to 7 weeks **(B)**. There are visibly more ^3^H-Thymidine positive SCs than those that were not dividing (^3^H-Thymidine—negative cells shown by arrows) after 7 weeks **(B)** as compared to 7 days **(A)**. **(C,D)** Immunostaining of SCs with S-100 identifies the dividing SCs (^3^H-thymidine positive SCs—arrows) and those that do not (^3^H-thymidine—negative cells shown by arrowheads). The proliferating S-100 positive SCs (arrows) are in close contact with the neurites of the DRG neurons. Scale bars = 30 μm.

It is known that denervated SCs proliferate a second time when regenerating axons make contact with the SCs in the denervated distal nerve stump (Pellegrino and Spencer, 1985). Five days after co-culture of denervated SCs with isolated DRG neurons and a 24-h exposure to ^3^H-thymidine, most of the 7-day acutely denervated S-100 positive SCs were labeled with ^3^H-thymidine and hence, had entered into the cell cycle (Figures 3A,D). Some of these SCs are identified in the phase-contrast autoradiograph with an arrowhead (Figure 3A) whilst those that were not dividing are identified by an arrow (Figure 3D). The proportion of mitotic SCs in the co-cultures of DRG neurons and SCs from the acutely denervated nerves was high, the ratio of the numbers of ^3^H-thymidine labeled and unlabeled SCs, expressed as a percentage, the proliferative index in Figure 2B, being ~75%.

The chronically denervated SCs from the left experimental hindlimb were also found to proliferate as shown in Figures 3B–F and quantitated in Figure 2B. However, the numbers of these ^3^H-thymidine positive SCs undergoing proliferation in the co-culture with DRG neurons declined significantly after chronic denervation (Figures 3B–F) with ~50% and ~30% of the SCs proliferating in the 7-week and 17-month chronically denervated nerves, respectively, the indices declining significantly with chronic denervation (*p* < 0.05; Figure 2B). The differences in proliferative indices for the SCs from the control 1 week and experimental 7 weeks and 17 months were all significant (*p* < 0.05). Note that the S-100 immunostaining of the SCs in Figure 3 revealed that the SCs were closely associated with and aligned along the neurites of the DRG neurons, irrespective of the duration of prior SC denervation *in vivo*.

### Chronically Denervated Schwann Cells Retain Their Capacity to Myelinate Axons

**Figure 4:**
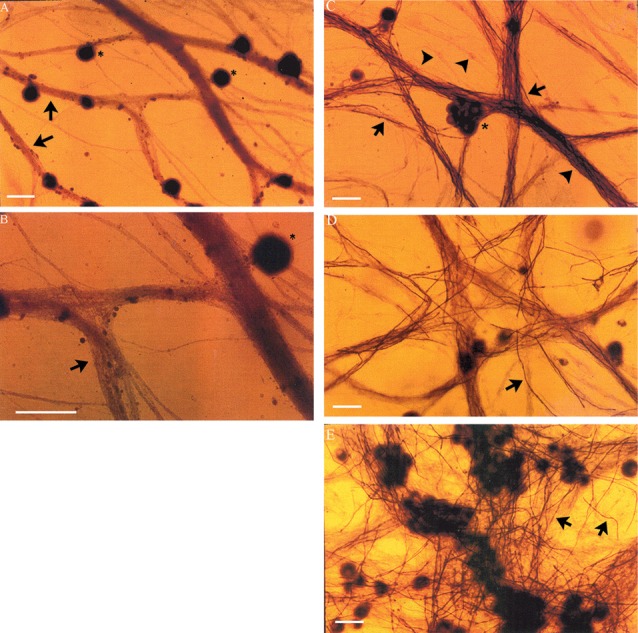
Neurites extended from DRG sensory neurons *in vitro* become myelinated in co-cultures with chronically denervated SCs. Myelination of the axons is not evident in the absence of SCs but it proceeds normally in SC-DRG sensory neuronal co-cultures, even after chronic SC denervation. **(A,B)** DRG neurons (*) maintained in culture for 24 days without SCs, were not myelinated as indicated by the arrows and seen by absence of Sudan Black staining in the unmyelinated axon bundles. Magnification was 20× **(A)** and 40× **(B)**. **(C–E)** DRG axons were myelinated when DRG neurons were co-cultured with SCs whether or not the SCs were acutely denervated (**C**: 7 days) or chronically for 7 weeks **(D,E)** or 17 months by transecting and ligating the sciatic nerve *in vivo* in the right and left hindlimbs, respectively, prior to co-culture. Scale bar = 30 μm.

DRG neurons (without non-neuronal cells) were cultured for 24 days with and without non-neuronal cells that were freshly isolated from denervated nerve stumps of the sciatic nerve and its branches after acute (7 days) and chronic (7 weeks, 17 months) denervation in the right and left hindlimbs, respectively. In the absence of added non-neuronal cells, the DRG neurons exhibited extensive axon bundles that were not stained with Sudan Black (Figures 4A,B), a lipid stain widely used for detecting and distinguishing lipid-rich myelin sheaths in tissue culture (Eldridge et al., 1987). This result clearly indicates that myelin was not formed by any residual SCs that might not have been detected in the pure neuron cultures (Figures 4A,B). In contrast, in the cultures of DRG neurons to which non-neuronal cells (isolated from acute or chronically denervated sciatic nerve stumps) were added, many elongated Sudan black-positive profiles were observed (Figures 4C–E). These profiles exhibited the well characterized structure of myelin sheathes. These findings demonstrate that the SCs that remain in chronically denervated nerve stumps retain their capacity to form myelin sheaths around neurites.

**Figure 5:**
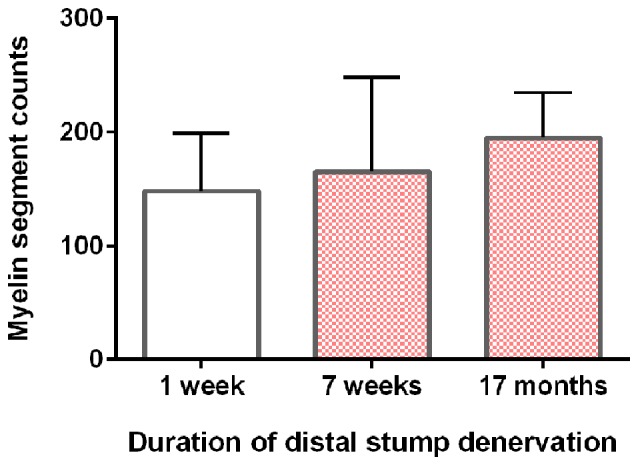
DRG sensory neurites *in vitro* become myelinated irrespective of the duration of SC denervation *in vivo*. The mean (± standard deviation) counts of myelin segments in SC-DRG co-cultures. There was no statistical difference in the counts when the co-cultures were performed with SCs that were acutely (7 days) or chronically denervated (7 weeks and 17 months) in the right and left hindlimbs, respectively (*p* > 0.05).

Neurite myelination was quantitated by counting the myelin segments formed at 24 days after the co-culture of DRG neurons with the acutely and chronically denervated non-neuronal cells (see “Materials and Methods” section). In the assay, the non-neuronal cells that were isolated from chronically denervated sciatic nerve stumps from the left hindlimbs (i.e., the 7-week and 17-month groups) and co-cultured with DRG neurons, elaborated as many myelin segments as was elaborated by those cells isolated from the 7-day (acutely denervated) nerves from the right hindlimbs. The mean values (± standard deviations) were not significantly different (*p* > 0.05; Figure 5).

## Discussion

This study demonstrated that first, SCs do survive in hindlimb nerve stumps that were chronically denervated for periods of up to 17 months. However, their numbers diminish dramatically. Second, the chronically denervated and atrophic SCs retain their abilities to associate with and to proliferate in response to contact with the neurites that extend from co-cultured DRG neurons. However, the rate at which the SCs re-enter the cell cycle is lower after a long period of denervation. Third, the chronically denervated SCs also sustain their capacity to myelinate the neurites extended in co-cultures of the cells with DRG neurons.

### Chronically Denervated SCs Atrophy and Many Die

Regeneration of injured peripheral nerves requires the presence of viable SCs in the denervated nerve stump (Hall, 1986). Orphaned SCs normally proliferate in response to the mitogenic factor(s) provided by ingrowing axons (Wood and Bunge, 1975; Salzer and Bunge, 1980; Salzer et al., 1980a,b; Sabue et al., 1984; Pellegrino and Spencer, 1985; Stewart et al., 1991; Morrissey et al., 1995). When SCs are chronically denervated for extended periods of time, the SCs undergo atrophy and decline in number (Weinberg and Spencer, 1978; Röyttä and Salonen, 1988; Salonen et al., 1988; You et al., 1997; Bradley et al., 1998; Dedkov et al., 2002; Jonsson et al., 2013; Kumar et al., 2016). There is a concomitant decline in the capacity of injured neurons, both motor and sensory, to regenerate their axons (Fu and Gordon, 1995a; Gordon et al., 2011; Jonsson et al., 2013), and in the numbers of regenerated nerve fibers (Jonsson et al., 2013). In our present study, the numbers of non-neuronal cells doubled 7 weeks after chronic distal nerve stump denervation in the experimental left hindlimb as compared to the number in the control right hindlimb (Figure 2A) but the cells likely included mast cells and fibroblasts that accumulate in the denervated stump (Salonen et al., 1988; Ronchi et al., 2017) which, in turn, complete their phagocytic activities within 6–8 weeks prior to withdrawing their processes (Röyttä and Salonen, 1988). SC nuclei in the distal nerve stump increase ~8-fold 25 days after a crush injury (Abercrombie and Johnson, 1946; Abercrombie and Santler, 1957) and to decline thereafter (Bradley and Asbury, 1970). Four weeks after chronic denervation, Salonen et al. (1988) reported an increase of 3.5-fold in SC number, suggesting that the number of proliferating SCs had already declined by 7 weeks. The macrophages that enter into distal nerve stumps within 3 days of denervation decline to low levels within a month (Gibson, 1979; Avellino et al., 1995) and therefore, are not likely to contribute to the non-neuronal cells that were enumerated after chronic denervation in the experimental nerves in the left hindlimbs (Figure 2A) nerve stumps in this study. That several non-neuronal cells enumerated after chronic denervation were likely to be SCs is supported by their S100 immunostaining (Figures 3D–F) after their co-culture with DRG neurons and hence, their exposure to endogenous mitogens on the surface of their neurites (Wood and Bunge, 1975; Salzer and Bunge, 1980; Salzer et al., 1980a,b; Sabue et al., 1984). These SCs demonstrated processes that were closely associated with and aligned along the neurites of the DRG neurons, irrespective of the duration of prior SC denervation *in vivo*.

The apparent discrepancy between the elevated non-neuronal cell numbers, of which most are likely to be SCs, and the reduced neuronal regenerative capacity at 7 weeks (Fu and Gordon, 1995a; Gordon et al., 2011) is likely due to the regression of the SCs to a non-supportive phenotype in which upregulated neurotrophic factors, including brain derived neurotrophic and glial derived neurotrophic factors, their p75 and GFRα-1 and GFRα-2 receptors, respectively, and soluble neuregulin 1 (Type I/II) and its Erb3/4 receptors, have declined back to low baseline values (Hoke et al., 2002, 2006; Boyd and Gordon, 2003; Gordon, 2015; Ronchi et al., 2017).

The non-neural cell numbers of >5 million at 7 days of acute nerve stump denervation in the right control hindlimb is dramatically lower, <2 million, in the right experimental hindlimb after 17 months of chronic stump denervation (Figure 2A), consistent with the progressive decline in numbers of viable SCs that were counted in rat hindlimb nerves after 3 and 6 months chronic denervation followed by 13 weeks nerve regeneration (Jonsson et al., 2013) and with the observed decline with light and electron microscopy, in 1–26-month chronically denervated rabbit nerves (Bradley et al., 1998), and 3–58-week chronically denervated rat nerves (Weinberg and Spencer, 1978). Microscopic observations demonstrated progressive Wallerian degeneration over the course of 7 weeks of chronic nerve stump denervation (You et al., 1997) with progressive disappearance of lipid-laden cells and residual myelin debris with cells of perineurial type (not fibroblasts) encircling small groups of denervated SC columns and progressive increase in the mass of collagen and elastin (Weinberg and Spencer, 1978; Bradley et al., 1998; see also Holmes and Young, 1942; Salonen et al., 1987a,b). The SC basement membranes progressively fragment, the shorter fragments dispersing throughout the endoneurium (Giannini and Dyck, 1990). It is likely that death of non-neuronal cells within chronically denervated nerve stumps explains the dramatic decline in their numbers. The nature of the death pathway is currently unknown and remains to be addressed.

### Chronically Denervated SCs Proliferate but Less so Than Acutely Denervated SCs

Despite high number of non-neuronal cells in the 7-week chronically denervated distal nerve stumps in the experimental left hindlimb, the S-100 positive SCs were less able than acutely denervated SCs in the control right hindlimb to proliferate in response to endogenous mitogens released by 5-day co-cultured DRG neurons (Figure 2B). Reduced capacity of p75-positive SCs to proliferate was reported by Kumar et al. (2016). At 1 week, ~75% of the acutely denervated SCs proliferated (Figure 2B), after which the proliferative capacity fell to ~50% and ~30% 7 weeks and 17 months after chronic denervation, respectively.

These findings of reduced proliferative capacity of chronically denervated SCs in the left hindlimbs contrast with earlier findings that SC proliferation was not altered by prior chronic denervation when the proliferation was evaluated in reinnervated nerve stumps, 13 weeks after surgical repair of 1–6-month chronically injured sciatic nerve stumps via an autograft (Jonsson et al., 2013). It must be recognized though, that, once the epineurium was removed from the excised distal nerve stump in that study, the ~1 mm cut nerve pieces were incubated in a SC growth medium for 2 weeks and that the medium was supplemented by the exogenous mitogens of forskolin and neuregulin NRG1 prior to the isolation and incubation of the SCs for 7 days (see also Kumar et al., 2016). That the proliferative capacity of the SCs within the 13-week reinnervated nerve stumps was the same after this procedure, irrespective of the period of acute or chronic SC denervation that preceded the 13 weeks of nerve regeneration (Jonsson et al., 2013), likely reflected a renewed capacity of the SCs contained within the distal stump to proliferate normally in response to regenerating axons. The demonstration by Casella et al. (1996) of significantly enhanced proliferation of isolated human SCs after their exposure to the same mitogens immediately after their isolation, as compared to that of the SCs isolated from untreated control nerves, provides further evidence that the proliferative properties of isolated SCs are altered after isolation and culture in media containing exogenous mitogens. In our experiments, proliferation of the SCs was analyzed only after the cells were exposed to the specific SC mitogens that are present on the surface of DRG neurites in the 5-day SC-DRG co-cultures (Wood and Bunge, 1975; Salzer and Bunge, 1980; Salzer et al., 1980a,b).

In the proliferation assay that we used, the isolated non-neuronal cells were labeled for 24 h with ^3^H-thymidine after SC-DRG co-culture for 5 days. The cells were then fixed and the dividing SCs identified as p75 immunoreactive (Figure 3). Therefore, the proliferation index measures the occurrence of new DNA synthesis and thereby, determines the capacity of the denervated SCs to enter into the cell cycle in response to the endogenous mitogens on the DRG neurons. SCs, whether immediately reinnervated or after a delay of 1–6 months, require time to re-enter the G1 phase of proliferation because significantly more reinnervated SCs proliferated at 5 as compared to 3 days after a 2-week period of incubation (Jonsson et al., 2013). Nonetheless, the reduced proliferation of chronically denervated SCs during the early phase of proliferation argue that the slower rate is unlikely to account for the findings.

The vigorous proliferation that we observed in acutely denervated SCs from the control right hindlimbs (Figure 2B) is consistent with the spontaneous upregulation of their erbB receptors following nerve transection (Carroll et al., 1997; Fricker and Bennett, 2011; Fricker et al., 2011; Chang et al., 2013) whilst their reduced proliferation with chronic denervation is likely linked to their downregulation of erbB2/erbB3 receptors (Li et al., 1997; Hall, 1999; Jonsson et al., 2013; Ronchi et al., 2017). The retained, albeit reduced, capacity of chronically denervated SCs to proliferate *in vitro* (Figure 2B) and for 5%–10% of freshly injured neurons to regenerate their axons through chronically denervated nerve stumps (Fu and Gordon, 1995a; Sulaiman and Gordon, 2000; Gordon et al., 2011), elaborate normal myelin sheaths, and to recover their normal size once target contacts are remade (Sulaiman and Gordon, 2000), demonstrate that the proliferative quiescence of the chronically denervated SCs from the experimental left hindlimbs is reversible: the dormant SCs re-enter the cell cycle and proliferate (Figures 2B, 3), albeit at a more sluggish initial rate.

### Chronically Denervated SCs Remyelinate Neurites

Sudan Black staining of the SCs isolated from chronically denervated distal nerve stumps of the experimental left hindlimbs and co-cultured with DRG neurons for 24 days, demonstrated normal capacity to remyelinate the neurites elaborated by the neurons (Figures 4, 5). Sudan Black staining of the lipid-rich membranes of compacted myelin sheaths was chosen to demonstrate remyelination of neurites rather than immunostaining because SC immunostaining without Sudan Black staining reveals SCs that are making myelin proteins but are not necessarily making myelin sheathes. SC immunostaining thereby, could give the erroneous impression that the SCs were making myelin sheaths when they were not. The *in vitro* findings of neurite myelination reported here, corroborate our *in vivo* findings of the remyelination of the low numbers of axons that injured neurons regenerate into chronically denervated nerve stumps (Sulaiman and Gordon, 2000). There were no significant differences in the extent of myelination by acutely or chronically denervated SCs derived from the right and left hindlimbs, respectively, whether the duration of chronic denervation was 7 weeks or 17 months (Figure 5). 1% to 5% of the DRG neurites were well myelinated, irrespective of the duration of the SC denervation *in vivo* prior to their co-culture, the remaining neurites being <1 μm in diameter and hence, unmyelinated. This “normal” myelinating capacity of chronically denervated SCs contrasts with the “severe impairment” of the myelination of the neurites of DRG neurons by 56-day denervated SCs after the non-neural passaging in the presence of mitogens and subsequent cell sorting (Kumar et al., 2016). This reported “severe impairment” however, does not correspond with the capacity of chronically denervated SCs to myelinate regenerated axons with restoration of the normal proportional relationship between myelin thickness and the size of the myelinated nerves (Sulaiman and Gordon, 2000).

### Summary

The survival and sustained capacity of chronically denervated SCs to re-enter the cell cycle together with the sustained capacity for these SCs to form myelin around neurites *in vitro* and regenerating axons *in vivo* indicates that surviving SCs may be stimulated to divide and support axon regeneration and re-myelination after chronic nerve injuries. Overall our results suggest that failure of axons to regenerate into long denervated nerve trunks is not due to lack of ability of the SCs to interact or respond to the axons. Rather, it is more likely to result from the reduced numbers of chronically injured neurons to regenerate their axons (Fu and Gordon, 1995b) and the extremely small number of SCs available to support and remyelinate the few regenerating axons in chronically denervated nerve stumps that and support nerve regeneration (Fu and Gordon, 1995a; Sulaiman and Gordon, 2000; Gordon et al., 2011). As such, cellular therapy may be a clinically viable option for patients who have sustained injuries to large nerve trunks.

## Author Contributions

All authors made equal contributions to the data interpretation and writing of the manuscript. PW and OS made the major contributions to the experimental execution.

## Conflict of Interest Statement

The authors declare that the research was conducted in the absence of any commercial or financial relationships that could be construed as a potential conflict of interest.
